# A Cre Mouse Line for Probing Irradiance- and Direction-Encoding Retinal Networks

**DOI:** 10.1523/ENEURO.0065-17.2017

**Published:** 2017-05-01

**Authors:** Shai Sabbah, Daniel Berg, Carin Papendorp, Kevin L. Briggman, David M. Berson

**Affiliations:** 1 Department of Neuroscience, Brown University, Providence, RI 02912; 2 National Institute for Neurological Disorders and Stroke, National Institutes of Health, Bethesda, MD 20892

**Keywords:** amacrine cells, bipolar cells, direction-selective ganglion cells, intrinsically photosensitive retinal ganglion cells, retina

## Abstract

Cell type-specific Cre driver lines have revolutionized the analysis of retinal cell types and circuits. We show that the transgenic mouse Rbp4-Cre selectively labels several retinal neuronal types relevant to the encoding of absolute light intensity (irradiance) and visual motion. In the ganglion cell layer (GCL), most marked cells are wide-field spiking polyaxonal amacrine cells (ACs) with sustained irradiance-encoding ON responses that persist during chemical synaptic blockade. Their arbors spread about 1 mm across the retina and are restricted to the inner half of the ON sublamina of the inner plexiform layer (IPL). There, they costratify with dendrites of M2 intrinsically photosensitive retinal ganglion cells (ipRGCs), to which they are tracer coupled. We propose that synaptically driven and intrinsic photocurrents of M2 cells pass through gap junctions to drive AC light responses. Also marked in this mouse are two types of RGCs. R-cells have a bistratified dendritic arbor, weak directional tuning, and irradiance-encoding ON responses. However, they also receive excitatory OFF input, revealed during ON-channel blockade. Serial blockface electron microscopic (SBEM) reconstruction confirms OFF bipolar input, and reveals that some OFF input derives from a novel type of OFF bipolar cell (BC). R-cells innervate specific layers of the dorsal lateral geniculate nucleus (dLGN) and superior colliculus (SC). The other marked RGC type (RDS) is bistratified, transient, and ON-OFF direction selective (DS). It apparently innervates the nucleus of the optic tract (NOT). The Rbp4-Cre mouse will be valuable for targeting these cell types for further study and for selectively manipulating them for circuit analysis.

## Significance Statement

Genetically modified Cre strains permit cell type-specific labeling and functional manipulations. We illustrate the utility of Rbp4-Cre mice for studying selected retinal cell types that encode the intensity of light and the direction of motion. Intensity encoding is thought to be uniquely mediated by intrinsically photosensitive retinal ganglion cells (ipRGCs). ipRGCs transmit intensity signals not only to the brain, but also intraretinally, partly through gap junctions to polyaxonal amacrine cells (ACs). We show that these ACs are tagged in Rbp4-Cre mice, confirm their persistent light responses under chemical synaptic blockade, and show that they costratify with, and are tracer coupled to, M2 ipRGCs. The line also labels a variant of direction selective (DS) RGCs, and a RGC that lacks intrinsic photosensitivity but encodes intensity.

## Introduction

The discovery and description of retinal cell types and circuits have been dramatically enhanced by the emergence of large numbers of strains of genetically modified mice with cell type-specific patterns of labeling. Of these, the most versatile are Cre lines, which permit diverse type-specific manipulations, including optogenetic and chemogenetic control. Here, we document the utility of a bacterial artificial chromosome (BAC) transgenic mouse, Rbp4-Cre, for studies of several inner retinal cell types. Some of these encode the irradiance (intensity) of light entering the eye. Among retinal ganglion cells (RGCs), such irradiance coding has been thought to be unique to those that are intrinsically photosensitive (ipRGCs). These ipRGCs all express melanopsin and respond directly to light but are otherwise diverse in form, physiology, and central projections (Sand et al., 2012). Two sublaminae of the inner plexiform layer (IPL) are strongly linked to ipRGCs. The first lies at the outer margin of the IPL, abutting the inner nuclear layer (INL). There, intermingled, lie the narrowly stratified dendrites of M1 ipRGCs, certain other ipRGC types, and dopaminergic amacrine cells (ACs). The second ipRGC-associated sublamina, containing most ipRGC dendrites, lies in the inner half of the ON sublayer, proximal to the ON cholinergic band. We show that all Cre-expressing ACs in the Rbp4-Cre line stratify within these same two laminae, and that at least one of them is coupled to ipRGCs there.

Early efforts to define the functional roles played by ipRGCs focused on their outputs to the brain, especially to the hypothalamic and brainstem circuits responsible for circadian synchronization and the pupillary light reflex. Spiking by ipRGCs encodes environmental light intensity and triggers reflexive circadian and pupillary responses. However, recent work has identified several “centrifugal” outputs, through which ipRGC signals are injected back into retinal networks. ipRGCs make both chemical and gap junctional contacts with other retinal neurons (Zhang et al., 2004; Zhang et al., 2007; Zhang et al., 2008; Müller et al., 2010; Reifler et al., 2015; Arroyo et al., 2016; Prigge et al., 2016) and ocular structures (Semo et al., 2014). They influence spontaneous retinal waves (Renna et al., 2011; Kirkby and Feller, 2013) and perinatal vascular development of the eye (Rao et al., 2013). Electrical coupling permits ipRGCs to inject excitatory currents into all ON displaced wide-field ACs (Reifler et al., 2015). Here, we demonstrate that in Rbp4-Cre mice, these ACs are tagged with enough selectivity to permit morphologic reconstruction, targeted patch recordings, and tracer-coupling studies. We confirm that a component of their light responses persists under chemical synaptic blockade, and show that they are selectively tracer coupled to the M2 ipRGC type, with which they costratify almost perfectly.

As a bonus, the Rbp4-Cre line also labels two distinct types of RGCs. The bistratified R-cell costratifies incompletely with the cholinergic plexuses, exhibits weak and inconsistent direction selectivity, and has some capacity for irradiance encoding. These cells project to the superior colliculus (SC) and a defined lamina of the dorsal lateral geniculate nucleus (vLGN and dLGN) distinct from that innervated by direction-selective (DS) cells. The other RGC, the RDS cell, is a bistratified DS RGC that responds transiently at both light ON and OFF. The RDS cell appears to innervate the nucleus of the optic tract (NOT).

## Materials and Methods

### Animals

All procedures were in accordance with National Institutes of Health guidelines and approved by the Institutional Animal Care and Use Committee at Brown University. We used adult mice (2-2.5 months old; either sex) of the strain Rbp4:Cre (031125-UCD, MMRRC). In some experiments, we used mice from crosses of the Rbp4-Cre line with the Cre-dependent tdTomato Cre-reporter mouse Ai14 (B6.Cg-*Gt(ROSA)26Sor^tm14(CAG-tdT^°^mat^°^)Hze/J^*) (The Jackson Laboratory).

### Intravitreal injections for Cre-dependent fluorescent labeling

Mice were anesthetized with isoflurane (3% in oxygen; Matrx VIP 3000, Midmark). A viral vector designed for Cre-dependent cell labeling with the mCherry fluorophore (rAAV2/Ef1a-DIO-hchR2(H134R)-mCherry; Vector Core, UNC; 1.5–2 μl of ∼1.5 × 10^12^ units/ml) was injected into the vitreous humor of both eyes through a glass pipette using a microinjector (Picospritzer III, Science Products GmbH). Animals were killed and retinas and brains harvested 14-21 d later.

### Tissue harvest and retinal dissection

Eyes were removed and immersed in oxygenated Ames’ medium (95% O_2_, 5% CO_2_; Sigma-Aldrich; supplemented with 23 mM NaHCO_3_ and 10 mM D-glucose). Under dim red light, the globe was cut along the ora serrata, and cornea, lens and vitreous removed. Four radial relieving cuts were made in the eyecup, the largest centered on the insertions of the lateral and medial recti, useful later as a reference axis. The other two were deliberately asymmetric (roughly dorsotemporal and ventral) to disambiguate retinal orientation. Using gentle suction, the retina was flat-mounted on a custom-machined hydrophilic polytetrafluoroethylene membrane (cell culture inserts, Millicell; Ivanova et al., 2013b) and secured in a chamber on the microscope stage. Retinas were continuously superfused with oxygenated Ames’ medium (32–34°C).

### Patch recording and dye filling of RGCs and ACs

Whole-cell patch-clamp current-clamp and voltage-clamp recordings of isolated flat-mount retinas were performed as described (Van Hook and Berson, 2010), using a Multiclamp 700B amplifier, Digidata 1550 digitizer, and pClamp 10.5 data acquisition software (Molecular Devices; 10 kHz sampling). Pipettes were pulled from thick-walled borosilicate tubing (P-97; Sutter Instruments); tip resistances were 5–6 MΩ when filled with internal solution, which, for current-clamp recordings, contained: 120 mM K-gluconate, 5 mM NaCl, 4 mM KCl, 2 mM EGTA, 10 mM HEPES, 4 mM ATP-Mg, 7 mM phosphocreatine-Tris, and 0.3 mM GTP-Tris, pH 7.3, 270–280 mOsm. For voltage-clamp recordings, the internal solution contained: 120 mM Cs-methanesulfonate, 5 mM NaCl, 4 mM CsCl, 2 mM EGTA, 10 mM HEPES, 4 mM ATP-mg, 7 mM phosphocreatine-tris, and 0.3 mM GTP-tris, pH 7.3, 270–280 mOsm. Green fluorescent dye (Alexa Fluor 488; Invitrogen) was added to the pipette for visual guidance under two-photon imaging and dye-filling for later morphologic characterization.

We pharmacologically blocked various receptors and pathways with one or more chemicals: (1) L-AP4 (100 μM; a Group III metabotropic glutamate receptor agonist; acting on the metabotropic glutamate receptor mGluR6); (2) D-AP5 (30 μM; an NMDA glutamate receptor antagonist); (3) DNQX (40 μM; an AMPA/kainate glutamate-receptor antagonist); or (4) ACET (1 μM; a selective GluR5-containing kainate-receptor antagonist). All were purchased from Tocris.

Whole-cell recordings and intracellular injections (described below) were conducted on a multiphoton Olympus FV1200MPE BASIC (BX-61WI) microscope equipped with a 25×, 1.05 NA water-immersion objective (XLPL25XWMP, Olympus) and an ultrafast pulsed laser (Mai Tai DeepSee HP, Spectra-Physics) tuned to 910 nm. Epifluorescence emission was separated into “green” and “red” channels with a 570-nm dichroic mirror and a 525/50 nm bandpass filter (FF03-525/50-32, Semrock, green channel) and 575-630 nm bandpass filter (BA575-630, Olympus, red channel), respectively. The microscope system was controlled by FluoView software (FV10-ASW v.4.1).

### Visual stimulation

Patterned visual stimuli, synthesized by custom software using Psychophysics Toolbox under Matlab (The MathWorks), were projected (AX325AA, HP) and focused onto photoreceptor outer segments through the microscope’s condenser (Borghuis et al., 2013). The projected display covered 1.5 × 1.5 mm; each pixel was 5 × 5 μm. The video projector was modified to use a single UV LED lamp (NC4U134A, Nichia). The LED’s peak wavelength (385 nm) shifted to 395 nm after transmission through a 440 nm short-pass dichroic filter (FF01-440/SP, Semrock), a dichroic mirror (T425lpxr, Chroma), and various reflective neutral density filters (Edmund Optics).

Quantum catches were derived from the stimulus spectrum (measured using an absolute-irradiance-calibrated spectrometer; USB4000-UV-VIS-ES, Ocean Optics) and spectral absorbances of mouse rod, cone, and melanopsin pigments (Govardovskii et al., 2000; Berson et al., 2002). At the highest light stimulus intensity, quantum catches were very similar among rods, cones, and melanopsin (∼13.5 log photons cm^−2^ s ^− 1^), independent of the cones’ relative expression of S- and M-cone pigments (Szél et al., 1992; Chang et al., 2013).

To study the cells’ ability to encode the absolute irradiance level, we used bright circular spots on a dark background (diameter, 1200 µm; Michelson contrast, 0.95, stimulus duration, 15 or 30 s; interstimulus duration, 15 s; four repetitions) at four or five irradiance levels at the plane of the photoreceptors (11-13.5 log photons cm^−2^ s^−1^). To assess directional tuning, we used a sinusoidal grating spanning two spatial periods (spatial frequency, 0.132 cycle/degree; Michelson contrast, 0.95; stimulus duration, 3.65 s; interstimulus duration, 5 s at uniform mean grating luminance) drifting in eight randomized directions (45° interval; drift speed, 4.5 °/s, four repetitions). To study receptive-field center-surround configuration, we used a series of seven circular spots of intermediate intensity (12 log photons cm^−2^ s ^− 1^) centered on the cell’s soma and covering a range of diameters (50-1200 µm). For RGCs, we used an additional set of seven spot diameters (50-400 µm) to sample smaller-spot responses more fully. For all stimuli, frames of the stimulus movie were brief but frequent, appearing for 50 µs during the short 185-µs interval between successive sweeps of the imaging laser. Response persistence allowed us to observe the evoked light responses although no stimulus was present during the interval of laser scanning and associated imaging (300 µs/sweep). The very rapid stimulus flicker (>2000 Hz) was well above critical fusion frequency in mice (Dai et al., 2015).

### Electrophysiology data analysis

Irradiance-response (IR) curves were calculated using the steady-state response, measured as the mean level over the five last seconds of the 15-s stimulus. The ability of cells to report the absolute irradiance was assessed by fitting the sigmoidal Naka-Rushton function (Naka and Rushton, 1966) to the cell’s steady state response to various stimulus irradiance levels (*R*):

$$R=R_{\max}\cdot 10^{\left(nE\right)}/(10^{\left(nE\right)}+10^{\left(nK\right)})$$

Where *R*_max_ stands for the cell’s predicted maximum response, *n* stands for the slope of the function, *E* stands for the irradiance measured in units of log photons cm^−2^ s ^− 1^, and *K* stands for the cell’s sensitivity.

The direction selectivity index (DSI) ranges between 0 (no direction selectivity) and 1 (maximal direction selectivity). It was calculated as (Kim et al., 2008):

$$\text{DSI}=\left|\sum_{\phi }r(\phi ){\text{e}}^{i\phi }\right|/\sum_{\phi }r(\phi )$$

where *r* is the response amplitude to stimuli moving at direction *Φ* (0°, 45°,…, 315°). The orientation selectivity index (OSI), which similarly ranges between 0 and 1, was calculated as:

$$\text{OSI}=\left(r_{\text{p}}-r_{\text{o}}\right)/\left(r_{\text{p}}+r_{\text{o}}\right)$$

where *r*_p_ stands for the response amplitude at the preferred orientation and *r*_o_ stands for the mean response at the two directions orthogonal to the preferred one (Zhao et al., 2013).

Before analysis, current and voltage traces were down-sampled to 0.1 kHz, and current traces were smoothed with a Gaussian filter (SD = 5). The response amplitude represented the peristimulus-time histogram (PSTH; for current-clamp recording under control conditions; bin width, 0.155 s), lower envelope of the voltage response (for current-clamp recording under any pharmacological manipulation that blocked voltage spikes), or current response (for voltage-clamp recording under any condition). All data were analyzed using custom Matlab scripts.

### Intracellular injections of Neurobiotin

For tracer-coupling analysis, selected tdTomato-positive ACs were injected with Neurobiotin. Fine glass pipettes (∼50 MΩ resistance) were filled at their tips with green fluorescent dye (Alexa Fluor 488; Invitrogen) and 4% Neurobiotin [N-(2-aminoethyl) biotinamide hydrochloride; Neurobiotin, Vector], and backfilled with K-gluconate-based internal solution (described above; Müller et al., 2010). A current pulse (-20 nA; 100 ms) triggered cell penetration. Fluorescent dye was iontophoretically injected (20-60 biphasic current pulses; -2000 pA for 500 ms and +500 pA for 400 ms) until dendrites were well-filled. Then, an additional set of current pulses of reversed polarity were applied to iontophoretically inject the Neurobiotin. Retinas were fixed (4% paraformaldehyde, 30 min, 20°C) and incubated overnight with streptavidin-Alexa Fluor 488 (S32354, Thermo Fisher Scientific) in 0.1 M PBS containing 0.3% Triton X-100 (Sigma-Aldrich). Anti-melanopsin and anti-ChAT immunostaining was conducted as described below. Filled cells were documented in z-stacks acquired in confocal (single-photon) mode.

### Immunohistochemistry of retina and brain

After recording, retinas were fixed (4% paraformaldehyde, 30 min, 20°C) and counterstained with one or more antibodies: (1) rabbit anti-cocaine and amphetamine-regulated transcript (CART; 1:1000; H00362, Phoenix Pharmaceuticals), a specific marker for most ON-OFF DS cells; Kay et al., 2011); (2) guinea pig anti-RNA-binding protein with multiple splicing (RBPMS; 1:1000; 1832-RBPMS, PhosphoSolutions), a pan-ganglion-cell marker; Rodriguez et al., 2014); (3) goat anti-ChAT (1:100, anti-ChAT; AB144P, Millipore); (4) rat anti-mCherry (1:1000, EST202, Kerafast); or (5) rabbit anti-melanopsin (1:1000, Advanced Targeting Systems).

### Reconstruction of retinal neurons and circuits by serial blockface electron microscopy (SBEM)

Tissue preparation and EM acquisition were performed as previously described (Ding et al., 2016). In short, a retina from an adult wild-type mouse (C57BL/6; postnatal day 30) was stained for EM while preserving the intracellular structure and details. A retinal block face (k0725) of ∼200 × 400 μm was imaged using a SBEM system. The incident electron beam had an energy of 2.0 keV and a current of ∼110 pA. Images were acquired with a pixel dwell time of 2.5 μs and size of 13.2 nm × 13.2 nm. The section thickness was set to 26 nm. Ten 112 consecutive block faces were imaged, resulting in aligned data volumes of 4992 × 16,000 × 10,112 voxels, corresponding to an approximate spatial volume of 50 × 210 × 260 μm^3^. The volume spanned the IPL and included parts of the ganglion cell layer (GCL) and INL. To facilitate viewing in KNOSSOS (http://www.knossostool.org), the dataset was split into cubes comprising 128 × 128 × 128 voxels.

The skeletons of cells were manually traced using the KNOSSOS annotation platform.

All skeletons were traced by at least two observers and any discrepancies resolved to ensure accuracy. We assigned bipolar cells (BCs) to established categories (Wässle et al., 2009; Helmstaedter et al., 2013; Kim et al., 2014) based on stratification level and lateral dimensions of the axonal arbor. Where we had dense sampling of neighboring BCs, the tiling pattern of arbors further aided the type assignment.

For each BC, we calculated the density of neurites as a function of IPL depth, where IPL 0% represents the INL-IPL boundary, and 100% represents the IPL-GCL boundary. We defined the INL-IPL boundary as lying at the level of the fifth percentile of neurite density of type 2 BC axon terminals, and IPL-GCL boundary at the 95th percentile of neurite density for rod bipolar axon terminals. We accounted for the slight tilt of the retinal laminae relative to the ultramicrotome cutting plane when calculating IPL depth. All analyses on skeleton data were performed using Matlab.

## Results

### Small subsets of amacrine and ganglion cells are marked in the Rbp4-Cre mouse

We revealed Cre expression in retinas of Rbp4-Cre mice by injections of Cre-dependent mCherry virus (AAV) or crosses with a tdTomato Cre-reporter strain (Ai14). In both cases, red fluorescence marked a minority of RGCs and ACs. BCs were also sparsely labeled, along with a subset of photoreceptors, but we have not studied these further.

**Figure 1.**
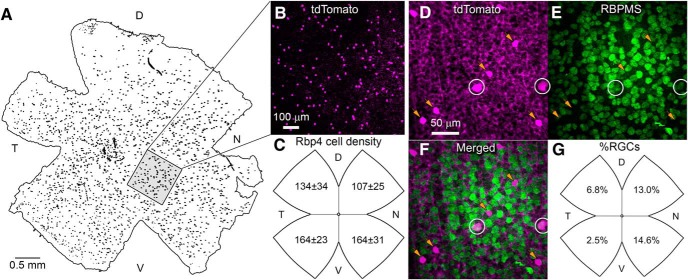
Density of Rbp4 ACs and RGCs varies across the retina. ***A***, Photographic montages of a representative retinal wholemount from a Rbp4-Cre;Ai14 mouse showing tdTomato labeling in Cre-expressing cell bodies in the GCL (black dots). Fluorescence image has been inverted and binarized for clarity. D, dorsal; N, nasal; V, ventral; T, temporal. ***B***, Magnified view of retinal region marked by black square in ***A***. tdTomato fluorescence is pseudocolored magenta; focal plane is in the ganglion-cell layer. ***C***, Density of tdTomato-positive cells in the GCL varies topographically and is lowest in the dorsal-nasal retina. Values for each quadrant are mean densities (cells/mm^2^) derived from counts in four fields, each 256 × 256 μm. ***D–F***, tdTomato-labeled cells of the GCL (***D***) include RGCs (white circles) identifiable by their RBPMS-immunolabeling (***E***) and displaced ACs (yellow arrows), which are RBPMS immunonegative. ***G***, Topographic variation in the percentage of tdTomato-positive cells that are RGCs.

In one fully mapped retina from an Rbp4-Cre;Ai14 mouse (Fig. 1*A*,*B*), there were 1893 tdTomato-positive cells in the GCL, corresponding to a mean density of 115 cells/mm^2^ (retinal area = 16.4 mm^2^). The density of labeled cells showed modest topographic dependence, being lowest in the dorsonasal retina (Fig. 1*C*). Labeled cells in the GCL comprised a mixture of RGCs and ACs. The RGCs were identifiable from their clearly labeled axons, traceable into the optic fiber layer, and from their immunoreactivity for the pan-ganglion-cell marker RBPMS (Rodriguez et al., 2014; Fig. 1*D–F*). However, most fluorescent cells of the GCL lacked both of these features and were thus displaced ACs. Approximately 90% of the fluorescent cells of the GCL (∼1700 cells in total) were displaced ACs, while the remainder (∼200 cells in total) were RGCs, as determined by RBPMS staining. The relative abundance of these cell classes varied topographically, with RGCs making up only 2.5% of the labeled cells in the ventrotemporal GCL, but 14.6% of them nasally (Fig. 1*D–G*).

**Figure 2.**
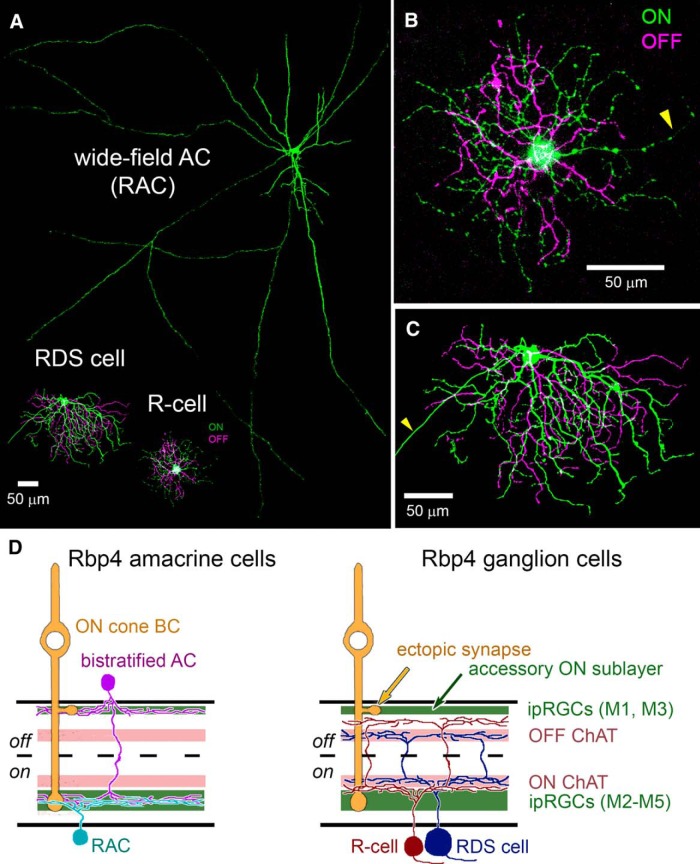
Morphology of Rbp4-Cre-positive cells of the inner retina. ***A–C***, Rbp4-Cre-positive cell types of the GCL. All images are maximum intensity projections showing the arbors as seen en face. To highlight the structure of the injected cell, images have been masked to hide extraneous fluorescence and pseudocolored to distinguish between processes in the ON sublayer of the IPL (green) from those in the OFF sublayer (purple). All cells were filled by intracellular dye injection after targeted recording of a tdTomato-positive RGC or AC in the Rbp4-Cre-tdTomato mouse. ***A***, Wide-field Rbp4-Cre-labeled displaced AC (RAC), shown to scale with the two RGC types, the RDS-cell and R-cell. ***B***, R-cell. ***C***, RDS-cell. Arrowheads in ***B***, ***C*** indicate axons. All scale bars, 50 μm. ***D***, Schematic diagrams illustrating stratification of Rbp4-Cre-labeled ACs (left) and RGCs (right) relative to ipRGCs and starburst ACs (ON and OFF ChAT bands). Polyaxonal displaced ACs (RACs, cyan) restrict their processes to the inner ipRGC plexus (lower green band). Bistratified ACs (purple), which have conventionally placed somas in the INL, stratify within both the outer ipRGC plexus (upper green band; the accessory ON sublayer, locus of ectopic synapses from ON cone BCs) and in the inner ipRGC plexus. R-cells (red), the most abundant Rbp4-Cre-labeled RGC type, deploy an outer arbor between the OFF ChAT band and the outer ipRGC plexus; their inner arbor stratifies near the inner boundary of the ON ChAT band. RDS-cells (blue) deploy their dendrites strictly within the ON and OFF ChAT bands.

Labeled RGCs were readily divisible into two distinct types. The first, more common type (here termed the R-cell) was bistratified with a relatively small soma and compact dendritic field (Fig. 2*A*,*B*). The second, rarer type (here termed the RDS cell) was also bistratified, but resembled classic DS cells. These had distinctly larger somas and, typically, asymmetric dendritic arbors (Fig. 2*A*,*C*). An additional AC type was labeled in this line. These had small somata in the conventional position for ACs (inner margin of INL) and a bistratified dendritic arbor. Figure 2*D* compares the patterns of stratification for each of these four types, and a fuller description of each follows.

### Rbp4-Cre labels a unique population of displaced polyaxonal ACs of the inner ON sublayer

**Figure 3.**
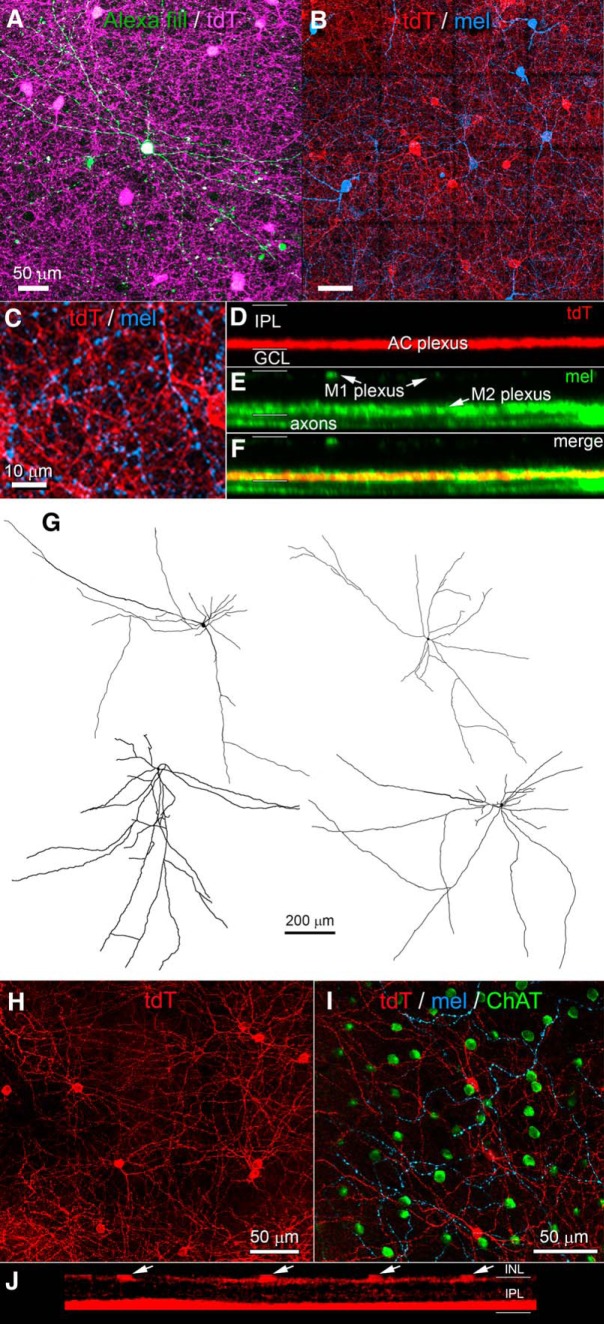
Morphology of ACs labeled in the Rbp4-Cre mouse line. ***A–G***, Morphology of RACs, the Rbp4-Cre-positive wide-field polyaxonal displaced ACs. RAC processes form a dense plexus in the inner ON sublayer intermingled with dendrites of M2 ipRGCs. ***A***, Single intracellularly dye-filled RAC (green) in relation to the dense plexus of fine, horizontally running tdTomato-positive processes in the Rbp4-Cre;Ai14 mouse (magenta). Maximum intensity projection of several optical sections through the GCL and inner ON sublayer of the IPL. ***B***, Relationship of the same plexus of wide-field ACs processes (red, tdTomato reporter for Rbp4-Cre) with the dendritic plexus of M2 ipRGCs (blue, anti-melanopsin). Tiled maximum intensity projection of ∼10 optical sections spanning the plexus and adjacent GCL but entirely proximal to ON ChAT. Scale bar, 50 μm. ***C***, Magnified subregion from ***B*** showing numerous points of potential contact between the two cell types. Scale bar, 10 μm. ***D–F***, Orthogonal projections of processes of Cre-positive ACs of the GCL (***D***), inner and outer ipRGC dendritic plexuses, marked by anti-melanopsin immunofluorescence (***E***), and a merged view (***F***) showing costratification of amacrine plexus with that of M2 melanopsin dendrites. ***G***, Drawings of single dye-filled RACs. ***H–J***, Cre-expressing wide-field bistratified ACs of the INL. ***H***, Somata and outer plexus of processes of these cells as shown in maximum intensity projection of a shallow z-stack of confocal optical sections spanning the INL-IPL border. ***I***, Single optical section at the level of this plexus in the most distal IPL showing the processes of tdTomato-labeled AC processes (red) with the dendrites of M1 ipRGCs (cyan), labeled by anti-melanopsin immunofluorescence. Somata of OFF starburst ACs (green) are also visible at this level. ***J***, Orthogonal maximal intensity projection of the z-stack from ***H***, showing the two plexuses of Rbp4-Cre-labeled ACs, an outer one (above) derived from the INL ACs (arrows) and the inner one (below) derived from both these INL ACs and RACs. Scattered labeling between these plexuses comprise connecting dendrites of the INL ACs. Somata in the GCL are not visible because the z-stack spanned only the IPL and proximal INL.

For convenience, we refer to Rpb4-Cre-positive displaced ACs (i.e., the labeled ACs in the GCL) as RACs. Their morphology is summarized in Figures 2*A*,*D*, 3 and Table 1. Cell bodies were relatively large for ACs (11.2 ± 1.4 μm in diameter; average ± SD; *n* = 46), comparable to those of neighboring starburst ACs and the smallest RGCs. They were multipolar and extended their processes horizontally within the inner ON sublayer of the IPL (Fig. 3*A*). There, they were quickly lost in a dense feltwork of labeled processes dominated by very fine varicose fibers following mostly straight trajectories (Fig. 3*A*, magenta, *B*, red). This plexus occupied the inner third of the ON sublamina (S4/S5), entirely proximal to (below) the ON cholinergic band. This plexus was matched almost exactly in depth with the inner plexus of melanopsin immunoreactive processes (Fig. 2*D*, left), consisting mainly of the dendrites of M2 ipRGCs (Fig. 3*C–F*; Berson et al., 2010). Intracellular dye injections of Cre-labeled RACs revealed their morphology in better detail (Figs. 2*A* and 3*A*, green, *G*). Their processes spanned roughly 1mm, encompassing about a fifth of the retina each (Fig. 3*G*; diameters 970 ± 130 μm; *n* = 4). The most widely spreading processes appeared axonal in form, having a uniform very fine caliber with periodic swellings, but most primary processes seemed dendritic, with a gradually tapering caliber as well as some spines and appendages. We therefore conclude that RACs are a form of wide-field polyaxonal AC (Fig. 2*A*).

**Table 1.** Summary (average ± SD) of morphologic properties of RGCs and ACs in the GCL of Rbp4-Cre mouse

	R-cell (*n* = 13)	R-cell EM (*n* = 1)	RDS (*n* = 4)	RAC (*n* = 4)
Soma diameter (μm)	12.9 ± 1.0 (*n* = 16)	16.1	17.5 ± 2.0 (*n* = 4)	11.2 ± 1.4 (*n* = 46)
ON dendritic field diameter (μm)	178 ± 35	192	177 ± 15	970 ± 130
OFF dendritic field diameter (μm)	138 ± 36	189	194 ± 27	
Global dendritic field diameter (μm)	186 ± 33	218	229 ± 6	
Total dendrite length (μm)	2912 ± 1076	3883	4965 ± 981	6765 ± 1595
Branch points	53 ± 15	47	95 ± 11	16 ± 5
Primary dendrites	5 ± 2	3	4 ± 1	5 ± 1
ON/OFF ratio of dendritic field diameter	1.3 ± 0.2	1	0.9 ± 0.1	
% of total branch points in inner IPL	61 ± 8	55	42 ± 3	
% of total dendritic length in inner IPL	62 ± 7	58	34 ± 6	

Soma diameters were estimated from photomicrographs of whole-mounted Rbp4-Cre;Ai14 retinas with exposures optimized for crisp definition of somatic profiles, thus avoiding overestimates from “bloom” of intense somatic fluorescence. Global dendritic field diameter represents the diameter of a circle that has the same area as a convex polygon minimally enclosing both inner and outer arbors.

### Rbp4-Cre labels a sparse population of conventionally placed bistratified ACs

A second type of Cre-labeled AC in this mouse line had conventionally placed somata in the INL (Figs. 2*D* purple, 3*H–J*). They were regularly spaced, suggesting possible mosaic organization (Fig. 3*H*), and of uniformly small size (diameter of 9.65 ± 0.55 μm, average ± SD, *n* = 23). Cre-dependent viral labeling revealed that their processes form a dense plexus in the outermost IPL, just proximal to their somata (Fig. 3*H–J*). Well-labeled cells almost invariably possessed a major dendritic process that descended through the IPL and branched, and apparently arborized, within the plexus of Cre-labeled RAC processes in the inner ON sublamina (Figs. 2*D*, left, 3*D*–*F*). We could not determine from such material the dimensions of the ON-sublayer dendritic fields of these bistratified ACs, nor what fraction of this inner amacrine-cell plexus was derived from these cells as opposed to RACs.

### RACs have sustained irradiance-encoding light responses

**Figure 4.**
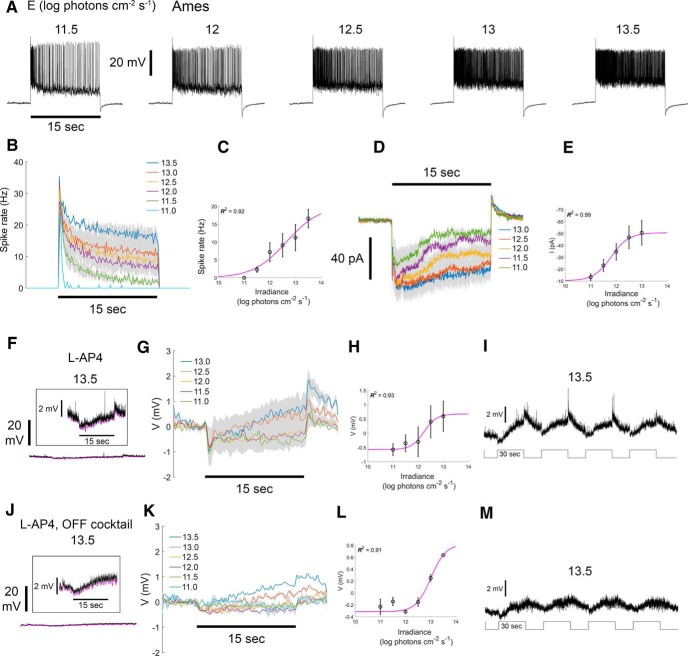
RACs have sustained irradiance-encoding light responses even under blockade of chemical synapses. ***A***, Voltage responses, including spiking, of a single RAC stimulated with a series whole-field light steps of various irradiances under control conditions (Ames’ medium). Whole-cell current-clamp recordings. Irradiance levels in log photons cm^−2^ s ^− 1^ are indicated above traces. Horizontal black bar below the leftmost trace indicates the timing of the 15-s stimulus (spot diameter, 1200 µm). ***B***, Average PSTHs of spiking evoked by light steps of various irradiances (*n* = 3 cells; average ± SEM). ***C***, IR curve based on data presented in ***B***. Data points and error bars indicate the average ± SEM of the steady state response, assessed during the last 5 s of the stimulus. Magenta line denotes the fitted sigmoidal Naka-Rushton function. The coefficient of determination (*R* ^2^) for the fit is indicated. ***D***, Excitatory inward currents evoked by a series of stimulus irradiance steps under control conditions. Whole-cell recording with voltage clamped at the chloride reversal potential. ***E***, IR curve derived from data in ***D***. ***F–I***, Effect of ON-channel blockade (bath applied L-AP4) on the voltage responses of RACs to light steps. Whole-cell current-clamp recordings. ***F–I***, Voltage responses of a representative RAC during ON-channel blockade. ***F***, Response to the brightest light step tested (black trace; 13.5 log photons cm^−2^ s ^− 1^). Lower trace is approximately to scale with those in ***A***. Inset, Higher gain display showing transients at onset and offset of the stimulus, plus a slow depolarizing drift of the membrane potential during the stimulus. The lower envelope of the voltage trace is shown in magenta. ***G***, Voltage responses at several irradiances (*n* = 3 cells; average ± SEM). Lower voltage envelope is shown, rather than raw voltage, to minimize distortion by spikes. ***H***, IR relationship based on data in ***G***. ***I***, Voltage responses to a series of four bright wide-field light steps each 30 s long. Note the gradual depolarization during the stimulus and slow poststimulus decay, which are characteristic of intrinsic melanopsin photoresponses in ipRGCs. ***J–M***, Effects of combined OFF and ON channel blockade on RAC light responses obtained in whole-cell current-clamp configuration (bath applied L-AP4 plus an OFF cocktail that included D-AP5, DNQX, and ACET; *n* = 3 cells), conventions the same as for matching plots in ***F–I***. Light still drives a slow depolarization, presumably due to persistence of melanopsin-dependent photoresponses in ipRGCs.

A recent study in rat retina found that all ON sustained wide-field displaced ACs are electrically coupled to ipRGCs (Reifler et al., 2015). The RACs we identify among the labeled Rbp4-Cre cells are similar morphologically to these rat ACs, so we suspected that RACs might have similar functional properties. Indeed, whole-cell current-clamp recordings confirmed this inference. Cre-labeled RACs were tonically depolarized, and increased their spiking in response to prolonged whole-field illumination (Fig. 4*A*). Responses included a transient peak that decayed within a few seconds to the steady-state level. Firing rates during this plateau phase varied as a function of stimulus irradiance. This is evident from spiking visible in individual voltage traces (Fig. 4*A*), from the average PSTH (average ± SEM; Fig. 4*B*), and from the Irradiance-Response (IR) function of pooled cells (Fig. 4*C*), derived from spike frequency during the five last seconds of the stimulus. We also examined the excitatory currents by voltage-clamping at the chloride reversal potential. Sustained excitatory inward currents persisted throughout the stimulus, with amplitudes correlated with stimulus irradiance (Fig. 4*D*,*E*). Light offset evoked an outward current (Fig. 4*D*) and modest hyperpolarization (Fig. 4*A*). These cells exhibited little evidence of an antagonistic surround (data not shown).

**Figure 5.**
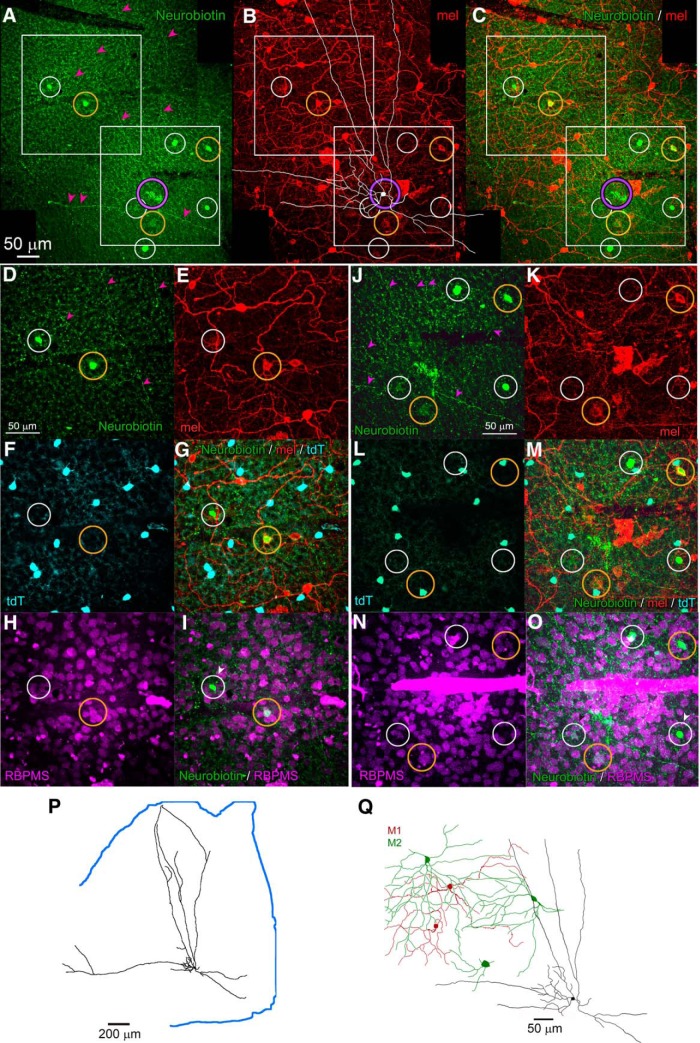
RACs are tracer coupled to M2 ipRGCs and other neurons of the GCL. ***A–C***, Intracellular dye filling and tracer coupling of a Rbp4-Cre displaced AC (RAC) in relation to melanopsin immunoreactivity. Montage of collapsed z-stacks (maximum intensity projections) covering a portion of the RAC’s arbor. ***A***, Fluorescent streptavidin labeling of Neurobiotin-injected RAC (purple circle; partly obscured by artifacts) and in eight cells tracer coupled to it (white and gold circles). Magenta arrowheads indicate the extremely slender processes of the dye-filled RAC. ***B***, Anti-melanopsin immunolabeling. A tracing of the filled RAC is overlaid in white. Circles around tracer-coupled cells are color coded for the melanopsin immunoreactivity of the marked cell: gold for immunopositive cells and white for immunenegative ones. ***C***, Merged view of ***A***, ***B***. ***D–I***, Identity of tracer coupled neurons in region marked by upper white square in ***A-****-****C***. Conventions as in ***A****–****C***. ***D***, Neurobiotin tracer. ***E***, Anti-melanopsin immunofluorescence. ***F***, Cre-dependent tdTomato labeling. ***G***, Merge of ***D–F***. ***H***, Immunofluorescence for the RGC marker RBPMS. ***I***, Merge of Neurobiotin (***D***) and RBPMS labeling (***H***). White arrowhead indicates a tracer-coupled cell that is both RBPMS immunenegative, and thus an AC, and Cre-negative and thus not a RAC. ***J–O***, Same cell as ***A–I*** but for the retinal region marked by the lower white square in ***A****–****C***. ***P***, Tracing of the Neurobiotin-injected RAC, which nearly encompasses this retinal quadrant; retinal margin marked by blue line. ***Q***, Drawings based on dendritic anti-melanopsin immunelabeling of three partially reconstructed M2 ipRGCs (green) that were tracer coupled to the Neurobiotin-filled RAC (black profile). Also traced are two M1 ipRGCs (red) in the same field that were not tracer coupled to the injected RAC.

To probe the origin of these light-evoked inward currents, we blocked the ON pathway by bath application of L-AP4 (an agonist of the Group III metabotropic glutamate receptors essential for light responses in ON BCs). ON-channel blockade dramatically reduced the light-evoked depolarization and firing (compare Fig. 4*F*, lower trace and Fig. 4*A*), but low amplitude responses persisted (Fig. 4*F*, inset). For bright stimuli (>10^12^ photons cm^−2^ s ^− 1^), these consisted of a rapid hyperpolarization (∼1 mV), followed by a larger, slowly developing sustained depolarization (Fig. 4*F*,*G*,*I*) that lasted at least 30 s, and decayed over many seconds after stimulus offset (Fig. 4*I*). Dimmer stimuli evoked only a very small sustained hyperpolarization (Fig. 4*G*). At light intensities above threshold for the sluggish depolarization, response amplitude increased with stimulus irradiance (Fig. 4*G*,*H*). This response is strikingly similar to melanopsin-driven responses of ipRGCs; it is depolarizing and irradiance encoding, with a high threshold (∼10^12^ photons cm^−2^ s^−1^), sluggish onset and slow poststimulus decay (Berson et al., 2002). Because these ACs are melanopsin-immunonegative (Figs. 3*B*, 5*G*), our findings parallel those of Reifler et al. (2015) in suggesting that the sluggish depolarizing response seen under these recording conditions derives from electrical coupling to ipRGCs.

RACs also appear to get modest excitatory input from the OFF channel. Blockade of the ON channel revealed a modest brisk depolarization at light offset (Fig. 4*F*,*G*,*I*). This was blocked (Fig. 4*K*,*M*) by supplementing the ON blocker L-AP4 with a mix of drugs designed to block the OFF channel. This “OFF cocktail” included D-AP5 (an NMDA glutamate receptor antagonist), DNQX (an AMPA/kainate glutamate receptor antagonist), and ACET (a selective GluR5-containing kainate receptor antagonist). The sluggish, irradiance-encoding, melanopsin-like response persisted under this more extensive blockade of chemical synaptic signaling (Fig. 4*J*,*K*,*M*).

### RACs are tracer coupled to selected ipRGCs and ACs

We used tracer coupling to test the hypothesis that the melanopsin-like response seen in RACs is attributable to gap junctional coupling to ipRGCs (Reifler et al., 2015). In Rbp4-Cre;Ai14 mice, two fluorescently tagged RACs were injected with Neurobiotin, which passes through gap junctions to label coupled cells. Both injected cells were confirmed to be wide-field ACs, and one of these is illustrated in Figure 5. Tracer-coupled cells labeled in these experiments included M2 ipRGCs, identified by their melanopsin immunoreactivity and characteristic dendritic arbors (Fig. 5*B*,*E*,*K*,*Q*). Also tracer-labeled were displaced ACs, identified by their small soma size, and their immunonegativity for either melanopsin or the RGC marker RBPMS (Fig. 5*H*,*I*,*N*,*O*). These ACs were not RACs, because they were not labeled by the tdTomato Cre reporter (Fig. 5*F*,*G*,*L*,*M*). A few tracer-coupled cells were melanopsin-immunonegative and weakly RBPMS immunopositive. It is unclear whether these are ipRGCs with weak melanopsin expression (M4 or M5 cells) or some other RGC types. We conclude that RACs form an extensive electrically coupled network comprising, at least, M2 ipRGCs and a distinct type of displaced ACs.

### Rbp4-Cre marks two types of bistratified RGCs

To assess the general morphology of Cre-expressing RGCs in the Rbp4-Cre line, we labeled large numbers of them through genetic crosses with the Ai14 tdTomato Cre-reporter mice or by Cre-dependent viral labeling. Melanopsin and ChAT immunolabeling provided laminar benchmarks. We could relatively easily trace the coarser dendrites of the RGCs through the thicket of amacrine-cell processes in the inner ON sublayer and thus reconstruct their arbors with reasonable confidence. Two clear types emerged: the smaller R-cell (Fig. 2*B*) and the larger RDS cell (Fig. 2*C*). Both were bistratified, but they differed in many other respects, as outlined below.

### Morphology of the R-cell

**Figure 6.**
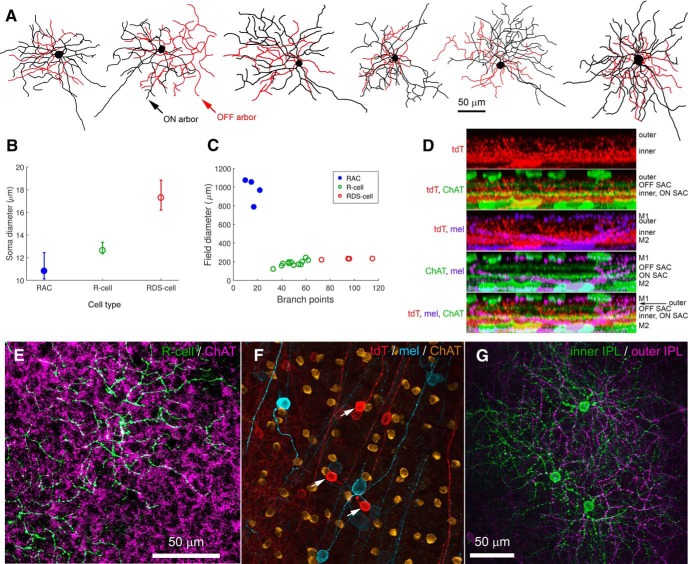
Morphology of the R-cell, a bistratified RGC labeled in the Rbp4-Cre line. ***A***, Examples of the common tdTomato-positive RGCs, R-cell, in the Rbp4-Cre mouse. ***B***, Variation in soma diameter across cell types in the GCL. Soma diameter differed significantly across the three cell types of the GCL [one-way ANOVA, *F*_(2,65)_ = 90.9, *p* < 0.001; *post hoc* pair-wise comparisons revealed that each cell type differed from the other two (*p* < 0.001)]. ***C***, Dendritic field diameter as a function of number of branch points for RACs, R-cells and RDS-cells. ***D***, Stratification of R-cell processes. Side-view maximum intensity projections of processes in Rbp4-cre cells (red) in relation to processes of M1 and M2 ipRGCs (purple; anti-melanopsin) and to starburst AC (SAC) processes (green; anti-ChAT). The fine Rbp4-cre-positive processes at the level of the inner melanopsin plexus (M2 ipRGC dendrites) are derived from wide-field displaced RACs; the coarse processes are derived mainly from R-cells, and stratify near and within the ON ChAT band and between OFF ChAT and the outer melanopsin plexus (M1 ipRGC dendrites). ***E***, Dendrites of the ON arbor of two virally labeled R-cells (green; flex AAV2-mCherry) tend to cofasciculate with the processes of ON starburst ACs (purple, anti-ChAT), which lie at or very near the same level as the R-cell ON processes. ***F***, Three closely spaced R-cells (red; white arrows) with visible axons, labeled in the Rbp4-Cre-tdTomato mouse. The other red cells are Rbp4-Cre-labeled displaced ACs (RACs). Shown for comparison are the somas and axons of ipRGCs (cyan, anti-melanopsin) and the somas of ON starburst ACs (gold, anti-ChAT). ***G***, The three closely spaced R-cells show a hint of mosaics formed by the dendritic arbors in both the ON (green) and OFF IPL sublayers (purple). Maximum intensity projection of several optical sections centered on the relevant arbor.

These RGCs were invariably bistratified. Their somas were small (diameter, 12.9 ± 1.0 μm); not much larger than those of starburst ACs, and smaller than those of M1 ipRGCs (Fig. 6*F*). Their dendritic fields were of intermediate size among murine RGCs, and their ON arbor (178 ± 35 μm in diameter) was typically slightly larger and more complex than the OFF arbor (138 ± 36 μm; Figs. 2*B*, 6*A*,*C*; Table 1). Although the two arbors were usually largely in spatial register, they were substantially offset from one another in a minority of cells. Local clusters of labeled R-cells had the appearance of fragmentary mosaics with well-spaced somas and a uniform plexus of moderately overlapping dendritic arbors in both the ON and OFF sublayers of the IPL (Fig. 6*F*,*G*). One tier of dendrites lay within the ON IPL sublayer, stratifying near the proximal margin of the ON ChAT band. These processes lay just distal to those of M2 ipRGCs and the wide-field displaced RACs described above (Fig. 6*D*). The ON tier loosely cofasciculated with the ON starburst processes (Fig. 6*E*). The OFF arbor was rather broadly stratified, filling the space between the plexus of OFF starburst AC dendrites and the plexus of M1 ipRGC dendrites marking the INL-IPL border (Figs. 2*D*, right, 6*D*).

### R-cells have sustained ON irradiance-encoding light-evoked responses

**Figure 7.**
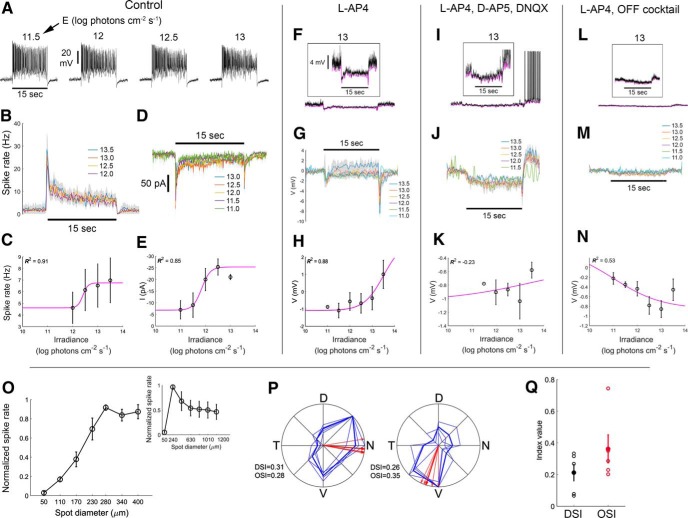
Rbp4 R-cells show sustained irradiance-encoding light-evoked responses. ***A***, Light-evoked firing, obtained in whole-cell current-clamp configuration, to a series of stimulus intensities (irradiance) under control conditions (retina bathed in Ames’ medium only). Irradiances in log photons cm^−2^ s ^− 1^ are indicated above traces. Horizontal black bar below the leftmost trace indicates the duration of a 15 s wide-field light step from darkness. ***B***, PSTHs averaged across cells (*n* = 4 cells; average ± SEM) for various irradiance levels. ***C***, IR curve based on data presented in ***B***. Data points and error bars indicate the steady state response (average ± SEM). The coefficient of determination (*R* ^2^) of the fit is indicated. ***D***, Light-evoked currents, obtained in whole-cell voltage-clamp configuration, to a series of stimulus irradiance levels under control conditions. The membrane voltage was clamped at the chloride reversal potential. ***E***, IR curve based on data presented in ***D***. ***F–N***, Pharmacological analysis of circuitry underlying R-cell light responses. ***F–H***, RGC responses, obtained in whole-cell current-clamp configuration, while blocking the ON pathway using L-AP4. ***F***, Example of a cell’s light-evoked voltage response (black trace) to the brightest stimulus tested (13 log photons cm^−2^ s ^− 1^). The lower envelope of the voltage trace is shown in magenta. Inset, Same trace at higher gain showing a sustained hyperpolarization in the voltage response as well as the lower voltage envelope throughout the stimulus duration. ***G***, Voltage envelope (*n* = 3 cells; average ± SEM) for various irradiance levels. ***H***, IR curve based on data presented in ***G***. ***I–K***, RGC responses (*n* = 5 cells), obtained in whole-cell current-clamp configuration, while blocking both the ON and OFF pathways using L-AP4, D-AP5, and DNQX. Conventions in individual plots are the same as in ***F–H***. ***L–N***, R-cell responses (*n* = 4 cells), obtained in whole-cell current-clamp configuration, after more complete synaptic blockade by further addition of the OFF channel blocker ACET to the L-AP4, D-AP5, and DNQX already in the bath. Conventions in individual plots are the same as in ***F–H***. ***O***, Firing rate in response to a series of bright spots on a dark background at varying sizes (main plot, 50-400 μm; inset, 50-1200 μm; *n* = 3 cells). ***P***, Example of direction and orientation selectivity tuning of two R-cells. Thick blue lines show the average cells’ response to sinusoidal gratings drifting in each of eight different directions, 45° apart. Thin blue lines show data from each of four single trials. N, nasal; D, dorsal; T, temporal; V, ventral. Red lines indicate the preferred direction based on four individual repetitions (thin lines), and their average (thick line). The DSI, OSI, and preferred direction (PD) of the cell are indicated. ***Q***, Average ± SEM DSI and OSI across tested cells (*n* = 5 cells).

R-cells exhibited sustained ON responses to whole-field illumination, and only a brief hyperpolarization at OFF (Fig. 7*A*). Higher light intensities resulted in reduced spike amplitude and regularity (Fig. 7*A*), presumably due to partial sodium-channel inactivation. The average firing rate (Fig. 7*B*) and the IR function derived from it (Fig. 7*C*) revealed only modest dependence of spiking on stimulus intensity. However, when clamping the membrane at the chloride reversal potential, the irradiance-dependence of the steady-state, light-evoked inward current became more apparent (Fig. 7*D*,*E*). Inward currents at light offset were also observed (Fig. 7*D*). The magnitudes of these, too, were irradiance dependent.

Blocking the ON channel with L-AP4 reversed the sign of the steady-state light response from depolarization and spiking (Fig. 7*A*) to hyperpolarization (Fig. 7*F*). Although the hyperpolarization was relatively modest, it was accompanied by a reduction in membrane noise, and a dramatic suppression of spiking (Fig. 7*F*). Both onset and offset of the light stimulus evoked a small, transient hyperpolarization. Membrane voltage varied only modestly as a function of stimulus irradiance (Fig. 7*G*,*H*).

We next supplemented the ON-channel blockade with agents designed to silence the OFF pathway (namely, a mix of NMDA and AMPA/kainate blockers, D-AP5 and DNQX). Although this strongly attenuated the transient hyperpolarizations at light ON and OFF, we were surprised to find that this incompletely blocked the sustained ON hyperpolarization, and that light offset induced poststimulus bursting in several cells (Fig. 7*I*). Membrane voltage was minimally related to stimulus irradiance under these conditions (Fig. 7*J*,*K*). We suspected that the residual light responses were due to incomplete kainate receptor blockade, so we added ACET (a selective antagonist for GluR5-containing kainate receptors). This rendered the cells largely irresponsive to light (Fig. 7*L–N*). Taken together, the data suggest that ON bipolar input normally dominates in R-cells, but that they also receive OFF bipolar input that is unmasked when the ON channel is silenced.

To study center-surround organization, we presented bright spots of various sizes (Fig. 7*O*). On average, the optimal spot diameter was 280 μm, which is substantially larger than the dendritic arbor. Larger spots attenuated the response, indicating the presence of an inhibitory surround (Fig. 7*O*, inset). R-cells generally showed some tuning for the direction or orientation of drifting gratings, but such tuning was inconsistent and typically weak (Fig. 7*P*,*Q*; DSI = 0.21 ± 0.13; OSI = 0.36 ± 0.22).

### Serial EM reconstruction of an R-cell and its synaptic inputs

To characterize the types of BCs that synapse onto R-cells, we mined a SBEM volume (k0725) generated from a wild-type adult mouse retina (Ding et al., 2016). The volume (50 × 210 × 260 μm^3^) spanned the IPL and the immediately adjacent portions of the GCL and INL. The retina had been processed so as to reveal intracellular structures such as synaptic ribbons and vesicles.

**Figure 8.**
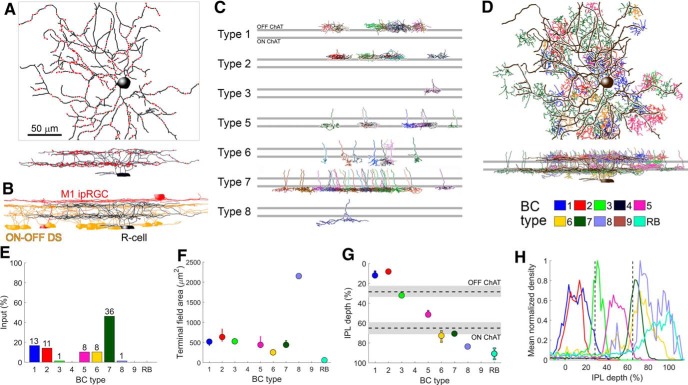
Serial EM reconstruction of an Rbp4 RGC and its synaptic inputs. ***A***, EM reconstruction of a presumptive Rbp4 R-cell (black) along with dyad synapses made onto it (red dots), as viewed en face (top) and in an orthogonal plane (bottom). Scale bar, 50 μm applies to panels ***A-****-****D***. ***B***, Orthogonal view of the presumptive R-cell (black) along with traces of several ON-OFF DS RGCs (yellow) and two M1 ipRGCs (red). The outer dendritic arbor of the presumptive R-cell stratifies below the M1 ipRGC plexus and just above the OFF ChAT band; its inner arbor stratifies just below the ON ChAT band. ***C***, Orthogonal view of BCs making identified ribbon synaptic contacts onto the presumptive R-cell. Top and bottom gray horizontal bars represent the OFF and ON ChAT bands, respectively. ***D***, EM reconstruction of a presumptive R-cell (black) along with BCs that synapse onto it, as viewed en face (top) and in an orthogonal plane (bottom). BC colors follow the scheme indicated in the key below. Note that the presumptive R-cell did not receive input from rod BCs (RB) or cone BCs of types 4 and 9. ***E***, Percentage of all bipolar input to the presumptive R-cell as a function of BC type. OFF input derived mainly through types 1 and 2, and ON input derived mainly through type 7. ***F***, Axon terminal field area (median and first and third quartiles) as a function of BC type. ***G***, IPL depth (median and first and third quartiles) as a function of BC type. ***H***, Mean normalized density of axonal processes as a function depth for each BC type. Although RB cells lacked synaptic contacts onto the presumptive R-cell, they were included in the analysis as a useful benchmark in the assessment of depth of axon-arbor stratification for BC types (see Materials and Methods).

Among many RGCs traced in the EM volume, we identified diverse bistratified forms. One of these RGCs closely resembled the R-cells as just characterized at the light microscopic level (compare Figs. 2*B*, 8*A* ). Several key morphologic measures obtained from the EM-reconstructed presumptive R-cell fell within 1 SD of the mean value of those measures for R-cells traced in confocal z-stacks (Table 1, compare R-cell and R-cell EM). The dendritic tree of the EM-reconstructed cell were similar in form to those of R-cells in branching structure, apparent dendritic field size and stratification (Fig. 8*A*). The outer arbor of the reconstructed R-cell stratified between the dendrites of reconstructed M1 ipRGCs and the OFF ChAT band (inferred from dendritic stratification of reconstructed ON-OFF DS cells). The inner arbor resided within and just below the ON ChAT band (Figs. 6*D*, 8*B* ). Although the soma diameter of this presumptive R-cell was larger than those of R-cells as assessed by confocal microscopy (16.1 vs 12.9 ± 1.0 μm), this might have resulted from osmotic factors during tissue fixation (Thorne and Nicholson, 2006; Mikula and Denk, 2015).

To identify the BC types that synapse onto the presumptive R-cell, we scanned its dendrites for ribbon synaptic contacts (Fig. 8*A*, red dots); subsequently, starting from randomly selected identified dyad synapses (128 out of 281), we reconstructed the complete axon and axon terminal of 76 BCs. To identify BC types, we assessed the stratification of their arbors in relation to one another and to the cholinergic bands (Fig. 8*C*,*G*,*H*). Figure 8*D* shows all reconstructed BCs synapsing onto the presumptive R-cell as projected onto the plane of the retina (D, top; en face view) or onto an orthogonal, vertical plane to show depth information (bottom). ON synaptic input to the presumptive R-cell was dominated by type 7 ON cone BCs, while its OFF synaptic input derived almost entirely from type 1 and 2 OFF cone BCs (Fig. 8*D*,*E*). Figure 8*F–H* quantify two features of axon terminals that distinguish bipolar types from one another, the dimensions of the terminal arbor (Fig. 8*F*) and their depth within the IPL (Fig. 8*G*,*H*). In general, the presumptive R-cell seemed to receive input from axon terminals of all BC types within its dendritic field, provided they costratified with the R-cell’s dendrites. ON inputs were more common than OFF inputs (78 vs 50 dyads in the inner and outer arbors; 53 vs 25 ON and OFF cone BCs), paralleling the dominant ON response observed physiologically (Fig. 7*A–E*).

### A novel BC type, cone BC type 0

**Figure 9.**
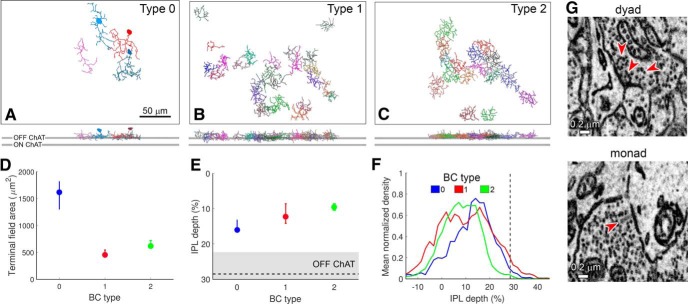
A novel OFF BC, type 0. ***A–C***, Partial mosaics of cone BC types 0, 1, and 2, as viewed en face (top) and in an orthogonal plane (bottom). Top and bottom gray horizontal bars represent the OFF and ON ChAT bands, respectively. Scale bar, 50 μm. The soma of one type 0 was not included in the SBEM volume, perhaps due to slight variation in the orientation of the retina within the SBEM volume. ***D***, Terminal field area (median and first and third quartiles) of type 0 BCs was larger than those of types 1 and 2. ***E***, IPL depth (median and first and third quartiles) of type 0 cells was larger than those of types 1 and 2. Gray horizontal bar represents the OFF ChAT band. ***F***, Mean normalized density of axonal processes of type 0 BCs was slightly more peaked and narrow than types 1 and 2. Vertical dashed line represents the OFF ChAT band. ***G***, Type 0 BCs made both dyad and monad synapses; arrowheads mark ribbons. All contacts onto the presumptive R-cell were dyads; the postsynaptic partners of monads are unknown.

Among the presynaptic neurons providing ribbon synaptic contact onto the presumptive R-cell, we identified four cells that appear to belong to a previously unknown BC type (Fig. 9). They superficially resembled types 1 and 2 OFF cone BCs but were clearly different from both. Most of the type 0 cells (three of the four) had cell bodies at the inner margin of the INL (the remaining cell’s soma lay outside the volume). This is a highly atypical position for BC bodies, but is characteristic of ACs. However, there is no precedent for synaptic ribbons in ACs, so for now we assume that this is a novel OFF cone BC type, which we provisionally term “type 0.”

Multiple features differentiated type 0 from type 1 (*n* = 25) or type 2 (*n* = 16) BCs. First, the arbors of type 0 BCs were sparsely branched and significantly broader than those of types 1 and 2 (Fig. 9*D*; 1614 μm^2^, 450 μm^2^, and 618 μm^2^ axon terminal field areas for types 0, 1, and 2; permutation one-way ANOVA, *p* < 0.001; by *post hoc* pairwise comparisons, arbor areas of type 0 were significantly larger than those of type 1 or 2, *p* < 0.001). Second, their axonal arbors stratified slightly more proximally (lower) in the IPL [Fig. 9*E*,*F*; IPL depth of 16%, 12%, and 9% for types 0, 1, and 2; permutation one-way ANOVA, *p* > 0.05; *post hoc* pairwise comparisons, type 0 axons arborized significantly deeper in the IPL than type 2 axons (*p* = 0.048), whereas the depth difference with type 1 axons did not reach statistical significance (*p* = 0.173)]. Finally, their arbors did not fit into the mosaics of BC type 1 and 2 axon terminal fields, and instead dovetailed with each other in a way that suggests an independent tiled mosaic in the IPL (Fig. 9*A–C*). Like other BCs, type 0 BCs make mainly dyadic ribbon contacts in the IPL.

### Morphology and physiology of RDS cells

**Figure 10.**
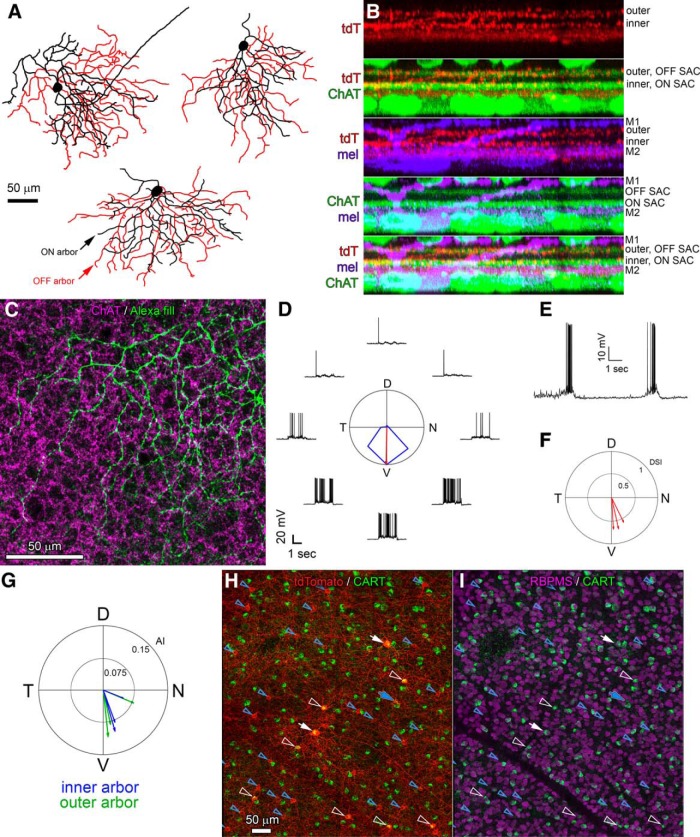
Morphology of DS RGCs in the Rbp4-Cre mouse. ***A***, Drawings of three DS RGCs labeled in the Rbp4-Cre mouse (RDS cells). ***B***, Dendritic stratification of an RDS cell. Five views of the same rotated orthographic maximum intensity projection derived from z-stacks taken in the vicinity of a single labeled RDS cell are shown. Views (top to bottom) show the individual tdTomato in the Rbp4:Cre;Ai14 mouse (tdT), tdTomato and anti-ChAT labeling (tdT/ChAT), tdTomato and anti-melanopsin labeling (tdT/mel), anti-ChAT and anti-melanopsin labeling (mel/ChAT), and tdT/mel/ChAT labeling. RDS dendrites with Rbp4-Cre-dependent viral labeling (red) match the depth of the ON and OFF ChAT bands [ON and OFF starburst AC (SAC) arbors, blue]. Both Rbp4 (red) and ChAT (green) labeling appear below the outer M1 ipRGC plexus and above the inner M2 ipRGC plexus (purple). ***C***, Dendrites of the inner arbor of a virally labeled RDS cell (green) cofasciculate with the processes of ON starburst ACs (anti-ChAT) which lie at or very near the same levels as the RDS-cell inner processes. Scale bar, 50 μm. ***D***, Voltage responses of a representative RDS cell to sinusoidal contrast gratings drifting in eight directions at 45° intervals. Polar plot shows response amplitude as a function of stimulus direction (normalized to maximum response). Red vector shows preferred direction in retinal coordinates (N, nasal; D, dorsal; T, temporal; V, ventral). ***E***, Voltage traces for the same representative RDS cell, illustrating the ON and OFF responses to the leading and trailing edges of a drifting bright bar. ***F***, A polar plot summarizing the direction preference (vector angle) and DSI (vector length). RDS cells preferred ventral motion on the retina. ***G***, A polar plot summarizing the direction of dendritic arbor asymmetry (vector angle) and asymmetry index (AI, vector length), for the inner (blue) and outer (green) arbors. ***H***, ***I***, Both types of Rbp4-cre RGCs (R-cells and RDS cells) are CART immunoreactive. Triple labeling in the GCL for Rbp4-Cre-tdTomato (red), CART (green; an immunomarker for ON-OFF DS RGCs), and RBPMS (magenta; an immunomarker for RGCs). Cre-positive ganglion cells are indicated by white markers. Most are R-cells (hollow white arrowheads), but two are RDS cells (filled white arrows). All of these are also immunopositive for RBPMS and CART (***I***). Cre-positive displaced ACs (RACs), indicated by blue markers, are identifiable from their RBPMS immunonegativity (***I***). Virtually all of these lack CART immunolabeling (hollow blue arrowheads), but one RAC appeared to be CART-immunopositive (solid blue arrow). Scale bar, 50 μm.

The other RGC type labeled in the Rbp4-Cre line, the DS cell here termed RDS, was rarer than R-cells, and easily distinguishable from them by virtue of its much larger soma (RDS: 17.5 ± 2 μm, *n* = 4; R-cells: 12.9 ± 1.0 μm, *n* = 16). RDS cells also had larger dendritic arbors. This was because the OFF arbor was larger in RDS cells (diameter, 194 ± 27 μm; *n* = 4) than in R-cells (138 ± 36 μm; *n* = 13); in contrast, ON arbor dimensions were very similar between the two types (RDS diameter, 177 ± 15 μm; R-cell diameter, 178 ± 35 μm). In branching structure and stratification, RDS cells strongly resembled other ON-OFF DS types (Figs. 2*C*, 10*A*; Huberman et al., 2009; Rivlin-Etzion et al., 2011; Trenholm et al., 2011; Dhande et al., 2013). Like those types, the RDS cell restricted its dendritic arbors to the two sublaminae containing processes of starburst ACs, revealed by ChAT immunolabeling (Fig. 10*B*). Moreover, like other ON-OFF DS cells, the RDS cell’s dendrites cofasciculated with the ChAT-immunopositive processes (Fig. 10*C*) and avoided the melanopsin immunoreactive plexuses at the margins of the IPL (Fig. 10*B*). We confirmed the direction selectivity of RDS cells by showing that they responded differentially to the direction of motion of sinusoidal gratings and bars. RDS cells were ON-OFF cells, responding to both the leading and trailing edge of the moving bar (Fig. 10*E*). Although our sample is small, all recorded RDS cells preferred ventral motion on the retina (Fig. 10*E*,*F*). They are thus reminiscent of cells labeled in the Hb9-GFP mouse line, which are also ON-OFF DS cells that prefer ventral retinal motion (Trenholm et al., 2011). Like Hb9-GFP cells, RDS cells exhibited dendritic asymmetry (Fig. 10*A*), with the center of the dendritic arbor displaced from the cell body in the cell’s preferred direction (compare Fig. 10*F*,*G*).

Like most ON-OFF DS types (Kay et al., 2011; Dhande et al., 2013), RDS cells were clearly immunoreactive for CART (Fig. 10*H*,*I*, white arrows). To our surprise, the R-cell, the other RGC type labeled in this line, was also virtually always CART-immunopositive despite its weak directional tuning (Fig. 10*H*,*I*, hollow white arrowheads). In the GCL, 97% of all RGCs (identified by their RBPMS labeling) that exhibited Cre-driven labeling in the Rbp4-Cre mouse also expressed CART (*n* = 64), whereas all but a handful of Cre-labeled displaced ACs (RACs; Fig. 10*H*,*I*, hollow blue arrowheads) were CART-negative. Two identified displaced Rbp4-Cre-labeled RGCs also lacked CART staining.

### Axonal projections of Rbp4-Cre RGCs

**Figure 11.**
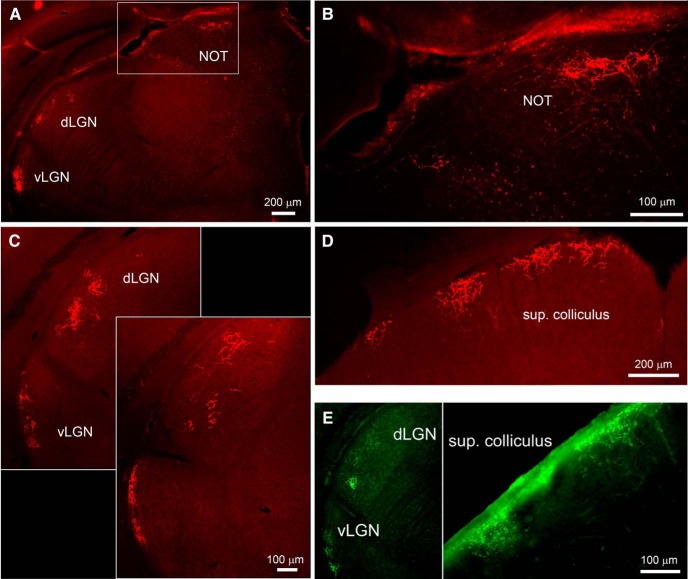
Brain projections of Rbp4 RGCs, and R-cells alone. ***A–D***, Axon terminal labeling following injection of a Cre-dependent viral reporter. ***A***, Labeling was evident in the LGN (***A***, ***C***) and NOT (***A***, ***B***). ***C***, Two coronal sections, consecutive along the rostral-caudal axis. Labeling was evident in the dorsal and ventral divisions of the LGN (dLGN, vLGN), but not in the intergeniculate leaflet (IGL). Labeling was restricted to a narrow band along the lateral margin of the vLGN. ***D***, Intense labeling was evident also in the superficial layers of the SC. ***E***, Axon terminal labeling following sparse labeling of Cre-expressing neurons. Only three RGCs were detectably labeled in this retina, and all were bistratified R-cells. Axon terminal labeling was detected only in the dLGN (at the boundary between core and shell regions), in the vLGN (left), and in the most superficial layers of the SC (right).

To study the central projections of RGCs in the Rbp4-Cre line, we made intraocular injections of Cre-dependent mCherry viruses. The advantage of this viral approach, as compared with crossing Rbp4-Cre mice with Cre reporter mice, is that it ensured that all fluorescent axons were of retinal origin. Labeled axon terminal fields were prominent in the dorsal devision of the lateral geniculate nucleus (dLGN). There, they were concentrated within a specific lamina, at roughly the transition between the external shell and main core of the nucleus (Fig. 11*A*,*C*), defined by their differential input from DS or melanopsin-expressing RGCs, respectively (Huberman et al., 2009; Ecker et al., 2010; Kay et al., 2011; Rivlin-Etzion et al., 2011). The superior colliculus (SC) was also prominently labeled, with highly branched fine axonal arbors that terminated only very superficially in the upper retinorecipient layers (Fig. 11*D*). Additional terminal fields were detected in a thin sheet at the lateral margin of the ventral LGN (Fig. 11*A*,*C*), and in the nucleus of the optic tract (NOT), a component of the accessory optic system (Fig. 11*A–C*). No labeling was detected in any other targets of retinofugal axons, including the medial terminal nucleus (MTN) and suprachiasmatic nucleus (SCN).

These axon terminal fields represent the combined projections of R-cells and RDS-cells, because the intraocular virus used transduced both RGC types, judging from their somadendritic architecture. However, in one case, viral transfection was sparse enough to label only three RGCs across the whole retina. All three were clearly R-cells. Their three axons were easily traced to the optic nerve head and into the brain. Three distinct arbors were evident in the dLGN, vLGN, and SC (Fig. 11*E*), but no axon terminals were detected in the NOT. These data suggest that R-cells innervate the LGN complex and SC, but not other retinorecipient targets, including the accessory optic system, while the RDS cells innervate at least the NOT.

## Discussion

Rbp4-Cre mice offer the opportunity to study and manipulate several interesting retinal cell types and circuits. These include a pair of wide-field AC types closely linked to melanopsin and ipRGCs; an ON bistratified RGC type (the R-cell) with weak direction and orientation selectivity and outputs linked to spatial vision; and a variant of the DS RGC class (the RDS cell).

The *Rbp4* gene codes for a retinol carrier protein. Because RACs are coupled to ipRGCs, which require retinoids for their intrinsic photosensitivity, we wondered whether the cells marked in the Rbp4-Cre mouse might be linked in some way to melanopsin phototransduction. However, pilot studies revealed no Rbp4-like immunoreactivity in the inner retina. Many transgenes are expressed in patterns unrelated to those of the targeted gene, presumably due to positional effects, and this may apply to the Rbp4-Cre BAC transgene in this mouse.

### RACs form a wide-field lateral inhibitory network coupled to ipRGCs

Two types of ACs are selectively marked in Rbp4-Cre mice. Both types contribute to the Rbp4-Cre plexus in the inner ON sublayer, where they costratify with the dendrites of most ipRGC subtypes (M2, M3, M4, and M5 cells). The other AC labeled in this mouse line, the conventionally placed bistratified type, has an outer dendritic arbor that intermingles with the dendrites of M1 and M3 ipRGCs.

RACs don’t merely costratify with ipRGCs; tracer coupling revealed that the processes of RACs and M2 ipRGCs make gap junctional contacts. This echoes the report of Pérez De Sevilla Müller et al. (2007), who observed dye coupling between M2 cells and displaced wide-field, monostratified polyaxonal ACs (PA-S5) that may match our RACs. RACs were also tracer coupled to melanopsin-immunonegative RGCs; these could be M4 or M5 ipRGCs, which often lack detectable melanopsin immunoreactivity (Ecker et al., 2010; Estevez et al., 2012). Indeed, Völgyi et al. (2009) found polyaxonal ACs tracer coupled to two RGC types; G_1_, which appears equivalent to M4 ipRGCs, also known as ON alpha cells (Estevez et al., 2012); and G_6_, which may correspond to M5 ipRGCs (Ecker et al., 2010). In addition to their coupling to RGCs, RACs were also coupled to Rbp4-Cre-negative displaced ACs. Therefore, RACs anchor a complex electrically coupled network in the inner ON IPL that encompasses multiple RGC types (including some ipRGCs) and at least one other AC type.

Our results provide several lines of evidence suggesting that RACs are influenced by ipRGC signals transmitted through gap junctions: (1) M2 ipRGCs are coupled through gap junctions to RACs (Fig. 5). (2) With chemical synaptic transmission intact, RAC spiking and excitatory currents are sustained and irradiance-encoding, mirroring those of ipRGCs (Fig. 4*A–E*). (3) Under blockade of chemical synaptic transmission, light triggers excitatory currents and increased RAC spiking, mirroring the responses of ipRGCs recorded under similar conditions. As in ipRGCs, these RAC responses are sluggish and sustained, and they persist long after the light stimulus is turned off (Fig. 4*J–M*). Presumably, under these conditions, intrinsic photoresponses of ipRGCs propagate into RACs through electrical coupling. (4) The response threshold of ipRGCs and RACs also match; under synaptic blockade, residual RAC light responses are evident mostly at the two highest light intensities tested, which are suprathreshold for driving the melanopsin-based phototransduction in ipRGCs (Fig. 4*J–M*). Therefore, RAC coupling to ipRGCs likely accounts for the persistence of RAC light responses under chemical synaptic blockade, and for the melanopsin-like sluggishness, insensitivity, sustained and irradiance-encoding nature of those light responses.

Reifler et al. (2015) were the first to describe such responses in displaced wide-field ON ACs in rat, and they proposed electrical coupling to ipRGCs as their basis. RACs resemble these rat ACs morphologically and may be homologous. Other possible equivalents are mouse polyaxonal ACs coupled to G_1_ and G_6_ RGCs (see above; Völgyi et al., 2009), WA4-1 cells (Lin and Masland, 2006), PA-S5 cells (Pérez De Sevilla Müller et al., 2007), and “Cluster 2” cells (Badea and Nathans, 2004). Like RACs, these are polyaxonal ACs with large displaced somata (12 μm in diameter), a relatively large, sparse dendritic arbor (∼450 μm in diameter) and wide-ranging axonal arbors (1-2 mm in diameter), both of which are restricted to the inner ON sublayer.

Residual RAC light responses were weak under synaptic blockade, but this probably understates the impact of electrical coupling *in vivo*. All ipRGCs, including M2 cells, exhibit robust light-evoked excitatory currents derived from BCs (Schmidt and Kofuji, 2010; Wong, 2012). These synaptically driven photic signals presumably pass through gap junctions to modulate AC excitability, just as melanopsin-driven photocurrents do. Synaptic blockade would eliminate this excitatory influence, along with direct bipolar drive to the RAC itself. Therefore, in the intact retina, RACs are likely to receive a mix of robust synaptically driven and intrinsic irradiance-encoding currents from M2 ipRGCs.

The wide-field architecture of RACs and their coupled partners provide a substrate for extensive lateral spread of locally generated irradiance signals. Spikes presumably transmit such signals throughout their sprawling polyaxonal arbors. Because RACs are almost certainly GABAergic, they probably inhibit target neurons in proportion to background light intensity, suggesting a role in network light adaptation. The postsynaptic targets of such inhibition are unknown, but could include ON cone BC terminals (types 6-9), dendrites of most ipRGC types (M2-M5), and many AC types (Helmstaedter et al., 2013).

Cre expression in RACs provides experimental opportunities. Cre-dependent calcium or glutamate indicators would facilitate functional imaging. Relatively selective optogenetic activation or silencing is also possible, though complicated by the diversity of Cre-expressing cells in this line. A favorable location for such studies would be the ventrotemporal retina, where RACs represent nearly all of the Cre-expressing cells in the GCL.

The bistratified morphology of the other AC type might predict ON-OFF light responses, but it could be purely ON. Its outer arbor narrowly costratifies with processes of M1 ipRGCs and dopaminergic ACs, both targets of ectopic *en passant* ON bipolar synapses in the OFF sublayer (Dumitrescu et al., 2009; Hoshi et al., 2009; Reifler et al., 2015).

### R-cell – an ON bistratified ganglion cell type

The R-cell is possibly a novel bistratified mouse RGC type. It has some features reminiscent of ipRGCs (sustained, irradiance-encoding ON excitatory drive) and others that link it to the DS system (weak directional tuning, CART-immunoreactivity, and partial costratification with starburst ACs).

R-cells join a variety of ON-dominant bistratified neurons of the GCL, including the M3 ipRGCs (Schmidt and Kofuji, 2011), ON-DS cells (Dhande et al., 2013), ON-orientation selective (OS) cells (Nath and Schwartz, 2016), and bistratified wide-field ACs (Reifler et al., 2015). We wondered whether R-cell outer arbors might be another target of the ectopic ON bipolar synapses in the outer OFF sublamina, but they were not. Serial-EM reconstruction revealed no ectopic ON bipolar contacts onto the R-cell outer dendrites, but abundant OFF bipolar input (Fig. 8), and this was reflected in their photoresponses (Fig. 7). Among RGCs, only ipRGCs have been thought capable of stably encoding light intensity, but we find R-cells do so to some degree, despite lacking melanopsin expression and intrinsic photosensitivity.

The responses of R-cells to a steady wide-field light stimulus differ from those of ipRGCs; yet they do share certain aspects. The responses of both R-cells and ipRGCs adapt over the first few seconds of the light stimulus, and then stay rather stable. In both cases, the response lasts at least 15 s, the longest light stimulus we used (Fig. 7*B*,*D*). The steady-state response of R-cells, expressed as a fraction of the maximum response, is smaller than for ipRGCs, and the dynamic range of the steady-state light response is much smaller for R-cells than for ipRGCs (R-cells: 18.5 pA; ipRGCs: 200-400 pA; data not shown). Thus, while R-cells encode irradiance (Fig. 7*E*), they do so over a relatively narrow range. On the other hand, the firing rate of R-cells did not show obvious irradiance-encoding (Fig. 7*C*). Thus, R-cell intrinsic mechanisms may largely temporally filter out the irradiance signal at the level of the cell’s spike train.

Bipolar inputs to R-cells were heterogeneous (ON: mainly from types 5 and 7; OFF: mostly from types 1 and 2). Our SBEM reconstruction also revealed contact from a novel OFF BC we call type 0. Like other OFF BCs, type 0 BCs’ axon terminals contain ribbons and stratify exclusively in the OFF sublayer of the IPL. However, they are distinguishable from type 1 and type 2 OFF cone BCs by axon terminal arbor dimensions and by mosaic analysis. Furthermore, they are distinguishable from type 3 and 4 OFF cone BCs based on stratification. type 0 BCs bear some resemblance to GluMI cells (Della Santina et al., 2016) and the presumably equivalent BC1B cells (Shekhar et al., 2016). Like type 0 BCs, both have a soma placed low in the INL, near AC somas, and an arbor stratifying roughly at the same depth as type 1 and 2 BCs. However, type 0 BCs have larger axonal arbors than GluMI/BC1B cells. Our SBEM volume omitted the outer retina, so we cannot say if type 0 BCs lack a process in the OPL, as GluMI and BC1B cells do.

R-cells resemble ON vertical OS RGCs (Nath and Schwartz, 2016). Both are bistratified in the same IPL sublaminae, have a larger ON than OFF arbors, lack marked dendritic-field asymmetry, have an ON-dominant response, and some sensitivity to stimulus orientation. However, R-cells have smaller dendritic arbors, a distinct temporal response profile to light steps, and substantially broader tuning for orientation than vertical OS cells.

Another possible R-cell equivalent is the medium-sized bistratified RGC (m-BGC), labeled in the PCP2-Cre mouse (Ivanova et al., 2013a). However, the two cell types slightly differ in soma diameter (R-cell versus m-BGC: 12.9 ± 1.0 vs 14.6 ± 1.6 μm), dendritic field diameters (ON: 178 ± 35 vs 219 ± 25 μm; OFF: 138 ± 36 vs 148 ± 24 μm), and ratio of ON to OFF dendritic field diameters (1.3 ± 0.2 vs 1.5 ± 0.1). Moreover, the inner arbor of R-cells which partly costratifies with the ON ChAT band, appears a bit more distal than that of m-BGCs. Also, m-BGCs exhibit many dendrites that ascend to the outer IPL before descending to the inner IPL; we rarely saw these in R-cells. Retinofugal projections in the PCP2-Cre and Rbp4-Cre mice are generally similar, though the heterogeneity of RGC labeling in both lines complicates any interpretation. Other possible morphologic equivalents of the R-cell include the G_12_ cell (Völgyi et al., 2009), type 8 RGCs (Helmstaedter et al., 2013), and the “V” RGCs (Sümbül et al., 2014), all of unknown physiologic type.

### RDS, a DS cell in the Rbp4-Cre mouse

The other RGC marked in this line, the RDS cell, is a variant of the ON-OFF DS RGC types. Superficially, the RDS cell resembles GFP-positive DS cells in the Hb9-GFP mouse (Trenholm et al., 2011). Like them, RDS cells prefer motion toward the ventral retina; they costratify and cofasciculate with both ON and OFF starburst processes in the IPL; they exhibit asymmetric ON and OFF dendritic arbors displaced from the soma toward the ventral retina; and they are CART-immunopositive (Kay et al., 2011; Trenholm et al., 2011). Additionally, the number of primary dendrites (4.0 ± 0.8 vs 3.1 ± 0.6), total dendritic length (4965 ± 981 vs 5371 ± 881 µm), and number of branch points (95 ± 11 vs 107 ± 31) do not differ significantly between RDS and Hb9 cells (Welch *t test* for unequal variances, primary dendrites: *t*= -2.09, df = 3.35, p = 0.12; dendritic length: *t*= 0.79, df = 3.47, p = 0.48; branch points: *t*= 1.26, df = 5.06, p = 0.26; n_RDS_=4, n_Hb9_=42). However, differences do stand out. Global dendritic field diameter (229 ± 6 vs 192.8 ± 2.7 µm) and soma diameter (17.5 ± 2.0 vs 12.7 ± 1.4 µm) of RDS cells are significantly larger than those of Hb9 cells (dendritic field diameter: *t*= -10.57, df = 6.71, *p* < 0.001; soma diameter: *t*= -5.23, df = 3.34, p = 0.01; n_RDS_=4, n_Hb9_=42; all morphologic statistics for Hb9 cells from Trenholm et al., 2011). Moreover, whereas several canonical ON-OFF DS subtypes are known to innervate the shell of the dLGN, RDS cells appear to project to the NOT, a part of the accessory optic system. It remains to be systematically tested whether the RDS cell represents a novel DS cell type, or instead, the same cell type marked in the Hb9-GFP mouse.

## Synthesis

Reviewing Editor: Siegrid Löwel, University of Goettingen Decisions are customarily a result of the Reviewing Editor and the peer reviewers coming together and discussing their recommendations until a consensus is reached. When revisions are invited, a fact-based synthesis statement explaining their decision and outlining what is needed to prepare a revision will be listed below. The following reviewer(s) agreed to reveal their identity: Johann Brandstatter, Karin Dedek

The mammalian retina contains more than 100 different cell types which form the building blocks of the diverse retinal circuits. While we have a good view of the photoreceptors, horizontal cells and bipolar cells, we lack knowledge on amacrine and ganglion cells which form the vast majority of retinal cell types (~80).With their study, the authors present an impressive piece of work and provide a detailed characterization of an amacrine cell and two ganglion cell types in the Rbp4-Cre mouse line and bring together morphological and physiological data. The data presented form a very good starting point for more sophisticated experiments aiming at revealing the cells' functions.

In general, the material presented in the paper is of high quality, the methods are sound, and the manuscript is well-written. The authors are to be congratulated on coming across this very useful Cre line with its sparse labeling of several retinal cell types that have thus far escaped the field's notice.

Apart from some minor comments, our main concern is - in line with earlier reviewers of the same manuscript - that the authors provide only correlative evidence that the characterized cells are related to irradiance encoding in the mouse retina. However, this seems only fitting for the RAC which most likely is identical to the spiking ON cells that Reifler et al. described in the rat retina to receive ON excitation from ipRGs via gap junctions. For the R-cells and in particular RDS the link to irradiance-encoding is rather weak. Therefore we strongly recommend to find a more general title such as: “A Cre mouse line for probing irradiance- and direction-encoding retinal networks”, or an even more general title such as: “Characterization of the morphology and light responses of three different retinal cell types in the Rbp4-Cre mouse line”. A title similar to the latter has the advantage of preparing the reader for a diverse collection of findings and reflects the content of the paper much better.

We also recommend to tone down conclusive statements about the identification of novel types of ganglion and bipolar cells.

Minor points:

- Methods: Please give some more details on the calculation of the PSTH. What was the bin size used? Were the data smoothed? Please give the concentrations for the blockers of synaptic activity.

- Figures 3, 5, 6: It would be very helpful to the reader if the authors labeled their immunostainings throughout the figures and always gave the respective marker for the immunostainings.
